# Improved Denitrification Performance of Polybutylene Succinate/Corncob Composite Carbon Source by Proper Pretreatment: Performance, Functional Genes and Microbial Community Structure

**DOI:** 10.3390/polym15040801

**Published:** 2023-02-05

**Authors:** Zhongchen Yang, Yanhong Lou, Hong Pan, Hui Wang, Quangang Yang, Yuping Zhuge, Jingying Hu

**Affiliations:** National Engineering Research Center for Efficient Utilization of Soil and Fertilizer Resources, College of Resources and Environment, Shandong Agricultural University, Tai’an 271018, China

**Keywords:** biodegradable polymer, degradation, polymer and composites, solid-phase denitrification, solid carbon source, pretreatment method, microbial community structure

## Abstract

Blending biodegradable polymers with plant materials is an effective method to improve the biodegradability of solid carbon sources and save denitrification costs, but the recalcitrant lignin in plant materials hinders the microbial decomposition of available carbon sources. In the present study, corncob pretreated by different methods was used to prepare polybutylene succinate/corncob (PBS/corncob) composites for biological denitrification. The PBS/corncob composite with alkaline pretreatment achieved the optimal NO_3_^−^-N removal rate (0.13 kg NO_3_^−^-N m^−3^ day^−1^) with less adverse effects. The pretreatment degree, temperature, and their interaction distinctly impacted the nitrogen removal performance and dissolved organic carbon (DOC) release, while the N_2_O emission was mainly affected by the temperature and the interaction of temperature and pretreatment degree. Microbial community analysis showed that the bacterial community was responsible for both denitrification and lignocellulose degradation, while the fungal community was primarily in charge of lignocellulose degradation. The outcomes of this study provide an effective strategy for improving the denitrification performance of composite carbon sources.

## 1. Introduction

Nitrogen pollution, which is mainly caused by superfluous inputs of nitrogen into receiving water, has seriously threatened ecological security. The threshold concentrations of total nitrogen (TN) and total phosphorus (TP) triggering cyanobacterial blooms were only 0.8 mg/L and 0.05 mg/L, respectively [1]. Hence, it is desirable to develop a facile and efficient nitrogen removal technique. As the most popular and cost-saving approach for nitrogen removal, biological denitrification has been widely used in wastewater treatment. Heterotrophic denitrification and autotrophic denitrification are two distinct types of biological denitrification, and heterotrophic denitrification uses organic carbon compounds as electron donors with a higher economy of scale and superior selectivity of end products [2]. Even so, incomplete denitrification due to a shortage of available carbon sources remains a huge challenge. Recently, solid-phase denitrification (SPD) based on solid carbon sources has been developed to be a promising alternative technique to solve the drawbacks of traditional water-soluble carbon sources [3,4]. The solid carbon sources in SPD are first hydrolyzed by extracellular enzymes excreted by microbes and then decomposed into soluble molecular organic substrates, thus being a vital factor affecting the performance of biological denitrification. Therefore, the biological degradability of solid carbon sources plays a crucial role in nitrogen removal [5].

Natural plant-like materials and synthetic biodegradable polymers are two kinds of solid carbon sources commonly used in SPD. Plant materials are low-cost and convenient with lower denitrification rates than synthetic biodegradable polymers, but the expensive denitrification costs also restrict the application of biodegradable polymers [3]. Therefore, the preparation of solid carbon sources with low cost, high bioavailability, and stable denitrification performance has become an inevitable puzzle. Intensive studies have demonstrated that blending biodegradable polymers with plant materials is an effective method to improve the biodegradability of solid carbon sources and save denitrification costs [6,7,8,9]. However, the recalcitrant lignin in plant materials is difficult to biodegrade and hinders the microbial decomposition of biodegradable cellulose and hemicelluloses [10], which might lead to the deterioration of denitrification performance and the wasting of resources. Various pretreatment methods for plant materials have been developed to intensify the biodegradability and accessibility of carbon sources [4,10,11,12]. The effects of pretreatment on the physicochemical structure of plant materials vary with different methods [13], which contributes to differences in the biological accessibility of available carbon sources and affects their electron supply capacity. In addition, the denitrification performance of solid carbon sources is prominently influenced by types of carbon sources, temperature, dissolved oxygen (DO), and pH [3]. Thus, it is necessary to determine the impacts of pretreatment and the interactions with these major factors on denitrification performance. In addition, the potential risks, including the excessive release of DOC, ammonium accumulation, and N_2_O emission, should also be noted.

Biological denitrification is conducted through a series of enzymatic reactions based on microorganisms with abundant metabolic types, which are capable of hydrolysis and denitrification. Hence, the analysis of the structure and function of the microbial community is conducive to understanding the mechanics of nitrogen removal and regulation in practical application. However, most studies have only focused on bacterial communities and ignored fungal communities, which have the ability to degrade lignocellulose with an efficient enzyme system [14]. The composite carbon sources rich in lignocellulosic and biodegradable polymers might create favorable conditions for the coexistence of bacteria and fungi. Therefore, how the bacterial and fungal communities interact with each other to achieve nitrogen removal and carbon supply needs to be revealed.

In the present study, corncobs pretreated by different physicochemical methods were blended with PBS to prepare composite carbon sources. Thus, the main objectives of this study were (1) to evaluate the effects of pretreatment methods on denitrification performance and potential risks; (2) to explore the effects of pretreatment degree, temperature, NO_3_^−^-N concentrations, and their interactions on nitrogen removal; and (3) to elucidate the interaction of microbial communities in the process of nitrogen removal and lignocellulose degradation.

## 2. Materials and Methods

### 2.1. Preparation of Composite Carbon Sources

The corncob powder of 100 mesh (0.12–0.15 mm, Jinan Hongrui Chemical Co. Ltd., Jinan, China) and biodegradable polymer PBS (cylindrical granules with diameter and height of 3–4 mm and molecular weight of 50,000–80,000 g mol^−1^, Shenzhen Huixin Plastic Chemical Co. Ltd., Shenzhen, China) were used to prepare composite carbon sources. The physicochemical methods adopted to pretreat corncob were acid pretreatment, alkali pretreatment, acid–heat pretreatment, and alkali–heat pretreatment. The corncob was immersed in dilute sulfuric acid (0.01 M) or sodium hydroxide solution (0.01 M) for 1 h to accomplish acidic or alkaline treatment. To achieve acid–heat treatment and alkali–heat treatment, the corncob immersed in dilute sulfuric acid (0.01 M) or sodium hydroxide solution (0.01 M) was heated at 120 °C for 1 h in an autoclave. All pretreated corncob was rinsed with distilled water and dried in an oven until constant weight; the unpretreated corncob was used as a control.

Thereafter, the biodegradable polymer PBS and pretreated or unpretreated corncob were blended with the weight ratio of 1:1 in a high-temperature internal mixer under 130 °C to prepare five kinds of composite carbon sources with a length, width, and height of about 1 cm, 1 cm, and 0.5 cm, respectively. According to the pretreatment methods, the composite carbon sources were named PC (PBS/corncob composite without pretreatment), PC-H (PBS/corncob composite with acidic pretreatment), PC-H-A (PBS/corncob composite with acid–heat pretreatment), PC-OH (PBS/corncob composite with alkaline pretreatment), and PC-OH-A (PBS/corncob composite with alkali–heat pretreatment).

### 2.2. Batch Experiment

To evaluate the denitrification performance of composite carbon sources, the batch experiment was conducted in a 250 mL conical flask including 10 g composite carbon source and 150 mL synthetic wastewater. All experiments were carried out in triplicate. During the inoculation period, 75 mL synthetic wastewater (30 mg L^−1^ NO_3_^−^-N, 6 mg L^−1^ PO_4_^3−^-P) and 75 mL activated sludge were fed into each conical flask, which was sealed with parafilm and cultured at 100 rpm min^−1^ and 25 °C in a constant temperature shaking incubator. After 2 days, the remaining solution was replaced with 150 mL synthetic wastewater (15 mg L^−1^ NO_3_^−^-N, 3 mg L^−1^ PO_4_^3−^-P), and updated every day. After the NO_3_^−^-N removal efficiency was stable, the composite carbon sources with superior denitrification performance were selected for the multi-factor experiment. The effects of pretreatment degree (0.0, 1.0, and 2.0), NO_3_^−^-N concentrations (5.0, 10.0, and 15.0 mg L^−1^), temperature (5.0, 15.0, and 25.0 °C), and their interactions on denitrification performance were estimated through response surface methodology (RSM). The analysis of data and model fitting were conducted to fit the relationship between responses and factors using Design-Expert 8.0 software, and the optimal values were obtained by reasonable value of the factors.

### 2.3. Sampling and Analytical Methods

The supernatant was sampled and filtered using 0.45 μm cellulose acetate membrane for measurement of NO_3_^−^-N, NO_2_^−^-N, NH_4_^+^-N, and TN [15]. DOC was determined using a TOC analyzer (TOC-C VPN 200 V, Shimadzu, Kyoto, Japan). pH was measured with a pH meter (PHB-4, INESA, Shanghai, China). Periodically, 10.0 mL of gas was extracted from the conical flask for N_2_O measurement via a gas chromatograph (GC-2010 Plus, Shimadzu, Kyoto, Japan). The Fourier-transform infrared spectroscopy (FTIR) spectra of fresh PC and fresh and used PC-OH were determined using a Fourier-transform infrared spectroscope (Nicolet is50, Thermo Fisher Scientific, Waltham, MA, USA).

### 2.4. DNA Extraction, Quantitative Real-Time PCR (qPCR), and Illumina MiSeq Sequencing Analysis

To investigate the effects of pretreatment methods on nitrogen functional genes and the microbial community, biofilm samples of composite carbon sources were collected at the end of the experiment. A microbial DNA extraction kit (Biocolors, Shanghai, China) was used for DNA extraction. The absolute abundance of the *16S rRNA*, ammonia-oxidizing archaea (AOA) *amoA*, ammonia-oxidizing bacteria (AOB) *amoA*, *amx 16S rRNA*, *nrfA*, *nirS*, *nirK*, *nosZ* I, *nosZ* II, *narG*, and *napA* genes was quantified using a BIOER real-time PCR system (9600 Plus, BIOER, Hangzhou, China) (Supplementary Materials). The qPCR assay was carried out in a volume of 20 μL, including 16.4 μL 2 × ChamQ SYBR Color qPCR Master Mix, 0.8 μL 5 μM forward primer, 0.8 μL 5 μM reverse primer, and 2 μL template DNA. For bacteria, the V3–V4 regions of the bacterial 16S rRNA gene were amplified with primers 338F (ACTCCTACGGGAGGCAGCAG) and 806R (GGACTACHVGGGTWTCTAAT). For fungi, the ITS1 region of fungi was amplified with primers ITS1F (CTTGGTCATTTAGAGGAAGTAA) and ITS2R (GCTGCGTTCTTCATCGATGC). To optimize the obtained sequences, the ambiguous and short sequences were removed. The remaining bacterial sequences were clustered into operational taxonomy units (OTUs) at a similarity threshold of 97% [16]. The purified fungal sequences were assigned to taxonomy using UNITE (https://unite.ut.ee/, accessed on 15 September 2022) databases.

### 2.5. Microbial Network Analysis

A co-occurrence network was constructed based on correlation coefficients and *p* values to show the interactions among the microbial community and environmental factors. To highlight the important interactions, only strong positive or negative relationships (absolute value of r > 0.6) and statistically significant (*p* < 0.05) were retained. Gephi software (https://gephi.org/, accessed on 28 September 2022) was used to visualize the network of the nodes and edges.

### 2.6. Statistical Analysis

All statistical analyses were performed using SPSS (version 26.0, IBM Corp., Chicago, IL, USA), and one-way analysis of variance (ANOVA) was used to identify the significance of the results. The results were considered to be statistically significant when *p* < 0.05.

## 3. Results and Discussion

### 3.1. Denitrification Performance, DOC Release, and N_2_O Emission of Different Composite Carbon Sources

**Figure 1:**
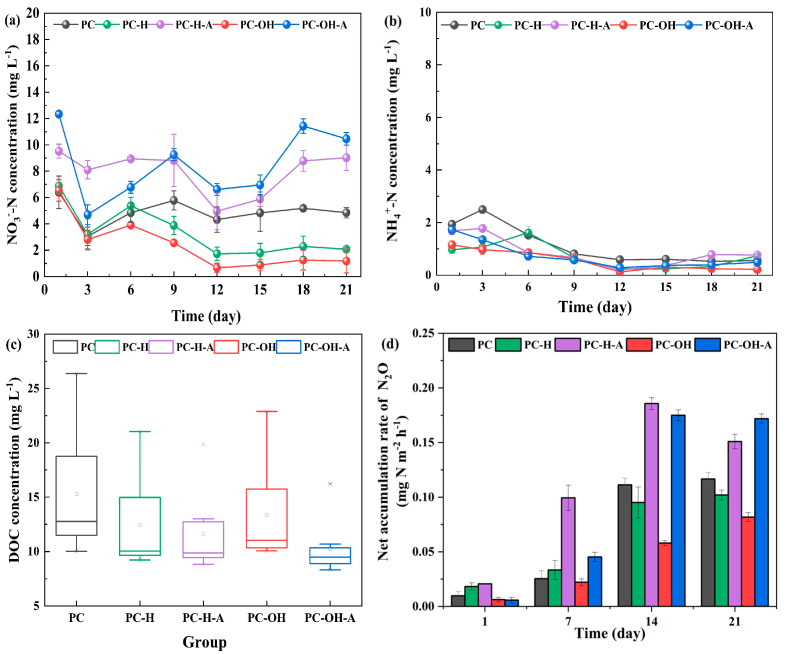
The variations of NO_3_^−^−N (**a**), NH_4_^+^−N (**b**), DOC (**c**), and N_2_O net accumulation rates (**d**) in different SPD systems. The hollow circles within each box represent the mean DOC concentrations, while the asterisks indicate the outlying data points.

The nitrogen removal performances of different composite carbon sources are depicted in Figure 1. The denitrification performance of composite carbon sources with different pretreatment methods presented remarkable differences. PC-H and PC-OH achieved better nitrate removal performances than PC after day 9, while the denitrification performances of PC-H-A and PC-OH-A were worse than those of PC throughout the experimental period. In the initial stage, denitrifying microorganisms consumed the readily biodegradable organic matter in composite carbon sources with high NO_3_^−^-N removal rates [4]. With the consumption of organic matter and biofilm formation, the NO_3_^−^-N removal rates gradually increased and finally stabilized, except for those of PC-H-A and PC-OH-A. After day 12, the average NO_3_^−^-N removal rate of PC-OH reached 0.13 kg NO_3_^−^-N m^−3^ day^−1^, which was significantly higher than that of PC-H (0.12 kg NO_3_^−^-N m^−3^ day^−1^) (*p* < 0.01) and PC (0.09 kg NO_3_^−^-N m^−3^ day^−1^) (*p* < 0.01). Compared to PC, the pretreatments of plant material obviously improved the NO_3_^−^-N removal rates of PC-OH and PC-H, which were similar to that of PBS/bamboo powder blends (0.13 kg NO_3_^−^-N m^−3^ day^−1^) [17] and higher than that of PBS/walnut shell blends (0.05 kg NO_3_^−^-N m^−3^ day^−1^) [18]. Blending biodegradable polymers with natural plant materials is an effective approach to improve bioavailability and reduce denitrification costs [8]. The denitrification rates of different composite carbon sources might be influenced by the intrinsic properties of carbon sources, operation conditions [19], and application modes [20]. Moreover, PC-H-A and PC-OH-A achieved optimal NO_3_^−^-N removal rates of 0.09 kg NO_3_^−^-N m^−3^ day^−1^ and 0.07 kg NO_3_^−^-N m^−3^ day^−1^, respectively (day 12), and then their NO_3_^−^-N removal rates gradually decreased. Hence, the pretreatment degrees of plant materials determined the denitrification performance of composite carbon sources to some extent, and excessive pretreatments might deteriorate denitrification performance.

NH_4_^+^-N accumulations in early operation are depicted in Figure 1b. The maximum NH_4_^+^-N concentration of 2.50 ± 0.10 mg L^−1^ was observed in PC (day 3), which was overtly higher than that of PC-H (1.62 ± 0.23 mg L^−1^, day 6) and PC-OH (1.16 ± 0.15 mg L^−1^, day 1). Thereafter, the NH_4_^+^-N concentrations gradually decreased and were maintained at 0.37–0.53 mg L^−1^ during the stable phase. The observed NH_4_^+^-N accumulations were mainly attributed to dissimilatory nitrate reduction to ammonium (DNRA), which resulted from the high C/N ratios in the initial period and were regulated by some strains from Desulfovibrionale, Bacteroidetes, and Planctomycetales [5,21].

The TN removal performance of PC-OH (49.06%, day 1) was distinctly superior to others, while PC-OH-A revealed the worst TN removal efficiency (6.51%) (Supplementary Materials). During the stable phase, the average TN concentration of PC-OH was 1.44 ± 0.53 mg L^−1^, which was remarkably lower than that of PC-H (2.73 ± 0.65 mg L^−1^) and PC (5.75 ± 0.57 mg L^−1^) (*p* < 0.05). The improvements in TN removal performance mainly resulted from the increased biodegradability of composite carbon sources [12]. However, the TN removal performances of PC-H-A and PC-OH-A significantly decreased from 60% (day 12) to below 40% (day 21), which demonstrated their unsustainable supply of available carbon sources.

The DOC release performances of different composite carbon sources are shown in Figure 1c. Compared with the original composite carbon source (PC, 15.31 ± 0.39 mg L^−1^), the DOC released from the pretreated composite carbon sources was significantly reduced (*p* < 0.01). Moreover, the amounts of organic matter released from PC-H and PC-OH were apparently higher than those of PC-H-A and PC-OH-A, respectively (*p* < 0.05), which confirmed that the pretreatments of plant materials distinctly decreased the DOC release of pretreated composite carbon sources. Although the DOC released from pretreated composite carbon sources largely decreased, the denitrification performances of PC-OH and PC-H were not largely affected. The main reason was that the stable supply of available carbon sources has been successfully achieved by denitrifying bacteria and fermentative anaerobic bacteria [22]. Moreover, the decrease in DOC release reduced the C/N ratios, which was unfavorable for DNRA organisms and conducive to alleviating the NH_4_^+^-N accumulation [23] (Figure 1b). Given a certain amount of carbon source, excessive pretreatment of plant material accelerated the consumption rates of carbon sources, which shortened the service life of the composite carbon sources and led to unsustainable denitrification performances. Therefore, the acid–heat pretreatment and alkali–heat pretreatment were not suitable to improve the denitrification performance of composite carbon sources.

Considering the potent greenhouse effect of N_2_O, the time profile of N_2_O net accumulation rates was measured (Figure 1d). The rates of N_2_O net accumulation in PC-H-A and PC-OH-A were notably higher than those of PC-H and PC-OH, respectively (*p* < 0.05), which indicated the occurrence of incomplete denitrification owing to insufficient supply of carbon sources. As the product of incomplete heterotrophic denitrification, the emission of N_2_O is affected by the amount and availability of carbon sources, C/N ratio, initial NO_3_^−^-N concentration, temperature, and pH [24,25,26]. In this study, the rates of N_2_O net accumulation might be mainly affected by the amount and availability of carbon sources caused by different pretreatment methods. The N_2_O emissions in PC-OH (day 21) accounted for 0.22% of the NO_3_^−^-N denitrified, which was higher than the results of Greenan et al. [27] (0.003–0.028%) but lower than that of Moorman et al. [28] (0.62%). Therefore, most of the NO_3_^−^-N denitrified in PC-OH was converted to N_2_ without significant N_2_O accumulation. Considering the denitrification performance, DOC release, and N_2_O emission, it is feasible to use PC-OH for the advanced nitrogen removal of wastewater with a low C/N ratio, and the mechanisms of nitrogen removal should be further explored for PC-OH.

### 3.2. Nitrogen Removal Performance, DOC Release, and N_2_O Accumulation Response to Variations of Different Factors

Models were constructed to explore the effects of pretreatment degree, initial NO_3_^−^-N concentrations, temperature, and their interactions on nitrogen removal performance using RSM. The models represented with coded factors and actual factors are as follows:

R_cod_ = 45.89 − 10.50A + 0.61B + 34.90C − 5.16AB − 9.36AC − 14.82A^2^ + 4.51C^2^ − 11.87A^2^C (1)

**Figure 2:**
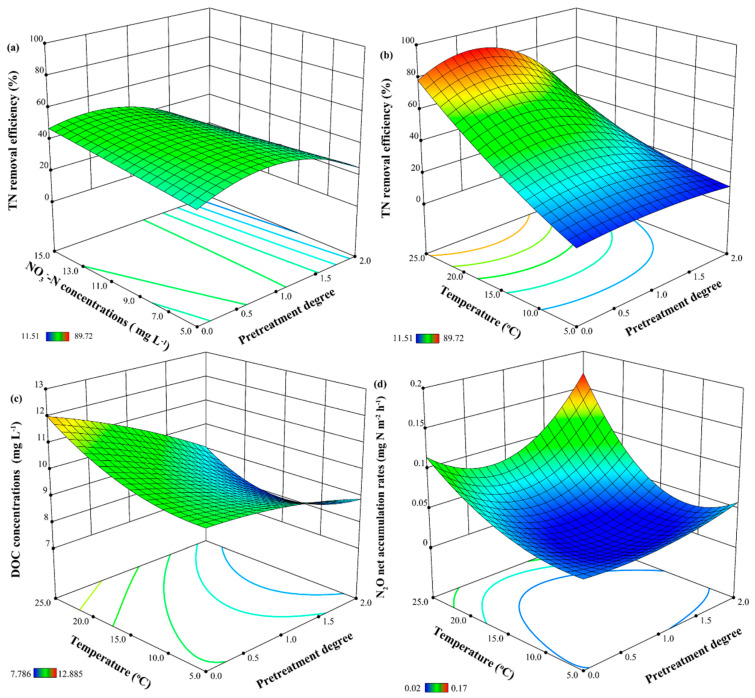
The simulated TN removal efficiency under the interactions of pretreatment degree and NO_3_^−^−N concentrations (**a**) and the interactions of pretreatment degree and temperature (**b**); the simulated DOC release under the interactions of pretreatment degree and temperature (**c**); the simulated N_2_O net accumulation rates under the interactions of pretreatment degree and temperature (**d**) using RSM.

R_act_ = −8.409 + 7.900A + 1.155B + 1.885C − 1.032AB + 1.438AC + 2.977A^2^ + 0.045C^2^ − 1.187A^2^C (2)

where R_cod_ represents coded TN removal efficiency (%), R_act_ represents actual TN removal efficiency (%), A represents pretreatment degree, B represents initial NO_3_^−^-N concentrations (mg L^−1^), and C represents temperature (°C). The model was significant with a *p*-value lower than 0.0001, and the lack of fit was not significant (*p* = 0.4763) (Supplementary Materials). The effects of the pretreatment degree and the interaction of pretreatment degree and initial NO_3_^−^-N concentrations on nitrogen removal performance were significant (*p* < 0.05) (Figure 2a). The TN removal efficiency increased with incremental NO_3_^−^-N concentration, while it first increased and then decreased with the increase in pretreatment degree. The TN removal performance was dramatically affected by the pretreatment degree, temperature, and their interaction (*p* < 0.01) (Figure 2b). The response of temperature to nitrogen removal performance was higher than that of the pretreatment degree, and the increase in temperature facilitated the improvement of the TN removal performance, which corresponds with the results of Shen et al. [29] and Hu et al. [30]. A total of 63 optimized TN removal efficiencies were obtained using RSM (Supplementary Materials); the highest predicted TN removal efficiency is 91.78% with the pretreatment degree of 0.53, the temperature of 25.00 °C, and the initial NO_3_^−^-N concentration of 15.00 mg L^−1^. Taking the practical operation into account, the composite carbon source would achieve the optimal TN removal performance (85.92%) with the pretreatment degree of 1.00, the temperature of 25 °C, and the initial NO_3_^−^-N concentration of 15 mg L^−1^, and this optimal TN removal performance is slightly lower than actual value (89.73%).

Likewise, the DOC release performance was distinctly influenced by the pretreatment degree, temperature, and their interaction (*p* < 0.05) (Figure 2c). The DOC concentrations increased with the increase in temperature but decreased with the increase in pretreatment degree. The rising temperature stimulates the activity of hydrolytic microorganisms, which contributes to the release of DOC. Pretreatment of plant material effectively improves the bioavailability of organic matter but also leads to a partial loss of organic matter, such as the cellulose and lignin dissolved in an alkali solution [31].

The N_2_O net accumulation rates were evidently influenced by the temperature and the interaction of temperature and pretreatment degree (*p* < 0.01) (Figure 2d). The N_2_O net accumulation rates increased with the increase in temperature, which was consistent with the results of Poh et al. [32] and Lee et al. [26]. In view of this, the nitrogen removal performance and DOC release were distinctly impacted by pretreatment degree, temperature, and their interaction, while N_2_O emission was mainly affected by the temperature and the interaction of temperature and pretreatment degree.

### 3.3. Characterization of PC-OH before and after Use

**Figure 3:**
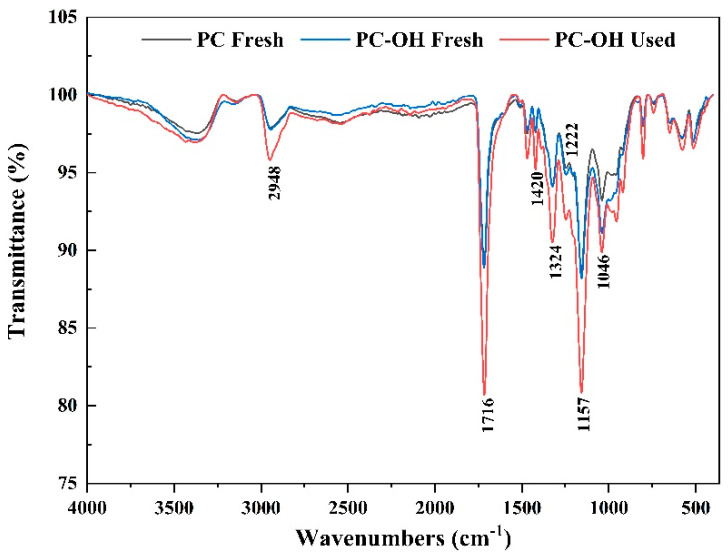
FTIR spectra of fresh PC and fresh and used PC-OH material.

Functional group changes of fresh PC and fresh and used PC-OH were observed by FT-IR spectroscopy (Figure 3). The fresh PBS had strong absorption peaks at 2948, 1716, and 1157 cm^−1^, which were assigned to -CH_3_ and -CH_2_ stretching and C=O (carbonyl) and C-O bonds of ester [33,34]. The absorption bands at 1222 and 1046 cm^−1^ were attributed to C–C plus C–O plus C=O stretch and C–O deformation in secondary alcohols, which were generally found in lignin. The evident decrease in the intensity of these peaks in PC-OH compared to those in PC suggested the effective removal of lignin in corncob [35]. Strong absorption peaks appeared at 1046, 1157, 1324, 1420, and 1716 cm^−1^, which corresponded to C-O-C asymmetric vibration of cellulose and hemicellulose, O-H blending of alcohol groups of carbohydrate, and aromatic skeletal vibrations with C-H in-plane deformation and -CH_2_ scissoring of lignin [36]. The attenuated peak intensity in used PC-OH verified the biodegradation of lignocellulose and PBS.

### 3.4. Nitrogen Functional Gene Analysis in Different SPD Systems

**Figure 4:**
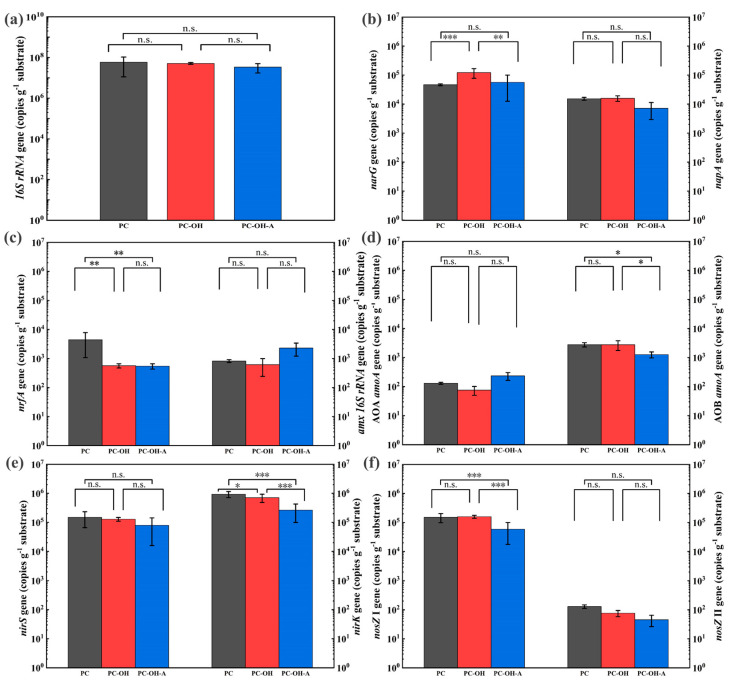
The absolute abundance of *16S rRNA* (**a**), *narG* and *napA* (**b**), *nrfA* and *amx 16S rRNA* (**c**), AOA *amoA* and AOB *amoA* (**d**), *nirS* and *nirK* (**e**), and *nosZ* I and *nosZ* II (**f**) on the surface of different composite carbon sources. The symbol *, ** and *** mean that the correlation is statistically significant at the 0.05, 0.01 and 0.001 level, respectively.

To further explore the involved nitrogen removal pathways, the absolute abundance of nitrogen functional genes was detected and is shown in Figure 4. The absolute abundance of the *16S rRNA* gene in PC (5.85 × 10^7^ copies/g) was higher than that in PC-OH (5.10 × 10^7^ copies/g) and PC-OH-A (3.37 × 10^7^ copies/g) with no significant difference, representing that the abundance of microorganisms in each SPD system had reached saturation. Most of the soluble small molecule organic substrates released from composite carbon sources are utilized by denitrifying microbes to supply denitrification, which is the most likely and favorite pathway [3]. The *narG* and *napA* genes encoding nitrate reductases are generally used as the markers of nitrate reduction. The abundance of *narG* genes was 3.07–7.86 times higher than that of *napA* genes, suggesting that nitrate reductases encoded by the *narG* gene dominated in NO_3_^−^-N reduction. Previous studies have demonstrated that the microorganisms containing the *narG* gene are more likely to live in anaerobic conditions, while the microorganisms harboring the *napA* gene prefer aerobic conditions [37,38]. Moreover, the notably higher amounts of *narG* genes in PC-OH (*p* < 0.05) indicated that the alkaline pretreatment of plant materials promoted the enrichment of denitrifying microorganisms and consequently improved denitrification performance.

Owing to the ubiquitous ammonia accumulation in the early stage of operation, the absolute abundance of the *nrfA* gene (the marker of DNRA) was determined (Figure 4c). DNRA occurring in SPD competes with denitrification and reduces NO_3_^−^-N to NH_4_^+^-N. PC acquired the obviously higher copy numbers of *nrfA* gene than others, which might be the main reason resulting in the maximum NH_4_^+^-N accumulation in PC (Figure 1b). The excessive soluble organic matter released from PC brought about a higher C/N ratio and created favorable reproduction conditions for microorganisms containing the *nrfA* gene [23]. Furthermore, the abundance of the *nrfA* gene was 1–2 orders of magnitude lower than that of the *narG* gene, manifesting that denitrification was superior to DNRA in nitrogen removal. Anammox bacteria play a major role in the nitrogen cycle by facilitating the conversion of NH_4_^+^-N and NO_2_^−^-N to N_2_ with the marker of the *amx 16S rRNA* gene. The quantity of *amx 16S rRNA* in PC-OH-A (2.31 × 10^3^ copies/g) was significantly higher than that in PC-OH (9.42 × 10^2^ copies/g) and PC (8.22 × 10^2^ copies/g), which revealed the more active anammox bacteria in PC-OH-A. During nitrification, ammonia-oxidizing archaea (AOA) and ammonia-oxidizing bacteria (AOB) engage in the oxidization of NH_3_ to NO_2_^−^ using ammonia monooxygenase enzymes [39]. The AOB *amoA* genes, ranging from 1.26 × 10^3^ copies/g to 2.78 × 10^3^ copies/g, were more abundant than AOA *amoA* genes (8.58 × 10 copies/g to 2.34 × 10^2^ copies/g) (Figure 4d), suggesting that AOB performed a dominant role in nitrification instead of AOA. The distribution and amount of AOA and AOB in different wastewater treatment systems may be affected by the characteristics of wastewater (NH_4_^+^-N and organic matter) and operation parameters (temperature, DO, and pH) [40]. The biofilm characteristics of the outer aerobic layer and the inner anoxic layer created the low-DO microenvironment in favor of AOB attachment [41].

Nitrite is reduced by nitrite reductase encoded by Cu-containing (*nirK*) and cytochrome cd1 (*nirS*). The distinct quantitative superiority gained by the *nirK* gene over the *nirS* gene (Figure 4e) suggested that the dominant nitrite reductase was cytochrome cd1- containing nitrite reductase expressed by the *nirS* gene, which was in accordance with previous studies [41,42]. The reduction of N_2_O is driven by nitrous oxide reductase, which is encoded by *nosZ* clade I or *nosZ* clade II. The evident enrichment of the *nosZ* gene in PC-OH was revealed with the maximum ratio of ∑*nos*/*16S rRNA*. Previous studies have shown that the quantitative balance between the N_2_O-producing microorganisms (*nirS* and *nirK*) and N_2_O-reducing microorganisms (*nosZ* I and *nosZ* II) regulated the net N_2_O emission to some extent [43], and the lower ratios of ∑*nir*/∑*nos* suggested the more complete denitrification with less N_2_O emissions [44]. In this study, the minimum ratios of ∑*nir*/∑*nos* in PC-OH (5.27) positively correlated with N_2_O net accumulation rates and corresponded to the results of Kong et al. [45] and Saarenheimo et al. [46]. Overall, the alkaline pretreatment of plant materials promoted the NO_3_^−^-N reduction and reduced N_2_O emissions by regulating the ratio of ∑*nir*/∑*nos*.

### 3.5. Microbial Community Structure

#### 3.5.1. Bacterial and Fungal Community Structure

**Figure 5:**
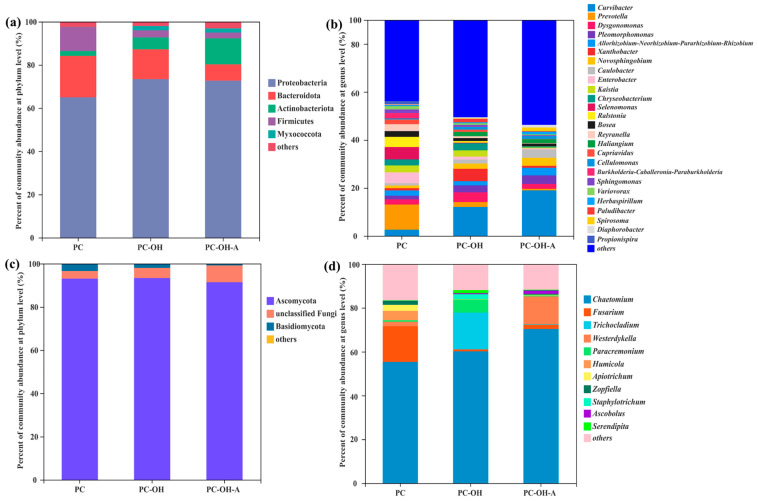
Bacterial community structures of the biofilms attached on different composite carbon sources at phylum (**a**) and genus levels (**b**); fungal community structures of the biofilms at phylum (**c**) and genus levels (**d**).

The structures of bacterial communities based on different composite carbon sources at phylum and genus levels (relative abundance > 1.00%) are shown in Figure 5. The Proteobacteria dominated in all biofilm samples, with relative abundances ranging from 64.98% to 73.45%, followed by Bacteroidetes (7.49–19.17%), Actinobateriota (2.27–12.04%), Firmicutes (2.72–11.07%), and Myxococcota (0.07–2.03%) (Figure 5a). Previous studies have demonstrated that most denitrifiers involved in SPD belong to the phylum Proteobacteria [3,6]. Bacteroidetes and Firmicutes could break down macromolecule substances, which accelerates the hydrolysis and utilization of biodegradable solid organic matter [47,48]. Myxococcota contain the functional genes for denitrification and were reported as the dominant organisms associated with partial denitrification [49]. At the genus level (Figure 5b), the relative abundance of *Prevotella* in PC was 10.50%, while it dropped to 2.06% and 0.64% in PC-OH and PC-OH-A, respectively. *Curvibacter*, the dominant genus in PC-OH (12.06%) and PC-OH-A (18.98%), accounted for only 2.60% in PC. It was reported that some abundant denitrifying organisms in activated sludge were affiliated with the genus *Curvibacter* [50]. *Prevotella* was one of the biomarkers used to infer the presence of potentially pathogenic microorganisms in aquatic environments [51], which might derive from inoculated sludge. Interestingly, the relative abundance of *Prevotella* decreased with the rising pretreatment degree. The genera *Allorhizobium-Neorhizobium-Pararhizobium-Rhizobium*, *Kaistia*, *Enterobacter*, *Sphingomonas*, *Chryseobacterium*, *Selenomonas*, *Ralstonia*, *Bosea*, *Haliangium*, *Cupriavidus*, *Burkholderia-Caballeronia-Paraburkholderia*, *Variovorax*, *Herbaspirillum*, *Diaphorobacter*, *Xanthobacter*, and *Paludibacter* were the main denitrifying bacteria attached on the surface of composite carbon sources (Supplementary Materials). Members of *Dysgonomonas*, *Reyranella*, *Cellulomonas*, *Propionispira*, *Pleomorphomonas*, and *Novosphingobium* have the ability to decompose recalcitrant organic compounds such as lignocellulose and polysaccharides, which could provide carbon sources for denitrifiers (Supplementary Materials). Some species of *Xanthobacter* and *Caulobacter* could metabolize organic materials and participate in the carbon cycle [52,53].

The dominant phylum was Ascomycota, with huge quantitative superiority (91.43–93.40%), followed by Basidiomycota (0.77–3.36%) (Figure 5c). Most of the denitrifying fungi that have been identified are affiliated with Ascomycota [54]. In addition, Basidiomycetes have the physiological capacity to degrade lignocellulose and xenobiotic compounds due to their oxidative enzymatic arsenal [55], which contributed to the biodegradation of lignocellulose in composite carbon sources. At the genus level (Figure 5d), *Chaetomium* played a leading role in biofilms, with a relative abundance of 55.36–70.32%. However, the second dominant genera were distinct and were *Fusarium* (16.26%), *Trichocladium* (16.78%), and *Westerdykella* (12.71%) for PC, PC-OH, and PC-OH-A, respectively. Some species of *Chaetomium* are known as cellulolytic fungi with the potential ability to degrade cellulosic waste [56]. *Fusarium* can produce diverse lignocellulose-degrading enzymes, which could be utilized for biotechnological applications [57]. *Trichoderma* can generate abundant lignocellulolytic enzymes and have been deemed as efficient compost microbes [58]. A previous study showed that *Westerdykella* exhibit the ability to degrade refractory organic compounds such as poly-ethylene terephthalate and polycyclic aromatic hydrocarbons [59]. Considering the lowest DOC level in PC-OH-A (Figure 1c), the excessive pretreatment degree of plant materials led to a large loss of organic matter and a lack of carbon sources, which eventually resulted in the enrichment of *Westerdykella* that could degrade refractory organic matter. In addition, most of the other classified genera (relative abundance > 1.00%) were able to degrade lignocellulose, such as *Paracremonium*, *Humicola*, *Apiotrichum*, *Staphylotrichum*, and *Ascobolus* (Supplementary Materials).

Based on the analysis of bacterial and fungal community structures, the bacterial community was responsible for both denitrification and lignocellulose degradation, while the fungal community was primarily in charge of lignocellulose degradation.

#### 3.5.2. Co-Occurrence Network Analysis for Microbial Communities and Environmental Factors

**Figure 6:**
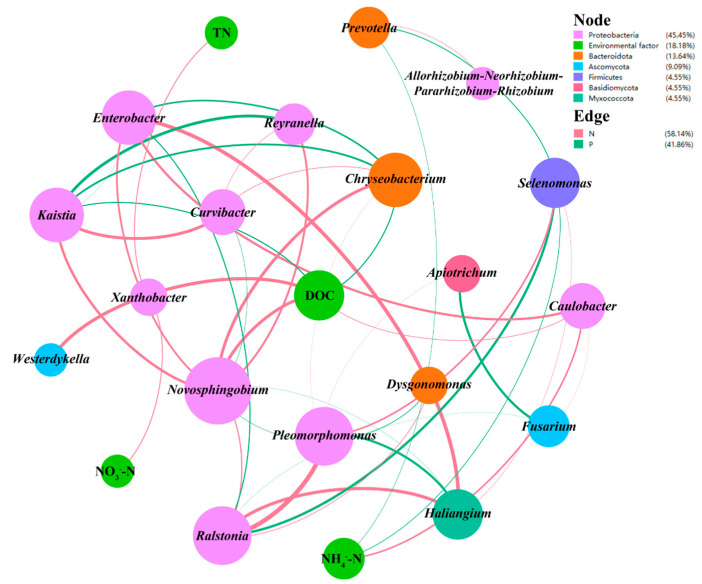
The co-occurrence network analysis between microbial genera and environmental factors. The nodes of unique genera are colored by phylum, and their sizes are proportional to the number of connections. The edges are weighted according to the Spearman’s correlation coefficient values. The negative and positive correlations are represented by red and green connections, respectively.

Co-occurrence network analysis is a valid method to simplify complex interactions among functional microbes, identify the keystone taxa, and infer potential relationships among microorganisms. To highlight the important and potential interactions, only strongly and statistically significant relationships were retained, which is beneficial for identifying key organisms in complex microbial communities. To explore the interactions between microbial genera and environmental factors, a co-occurrence network was constructed (Figure 6). A total of 18 genera and 4 environmental factors were involved in the network. The genus nodes belonged to four bacterial phyla (Proteobacteria, Bacteroidota, Firmicutes, and Myxococcota) and two fungal phyla (Ascomycota and Basidiomycota), and more than half of them came from Proteobacteria and Bacteroidota. *Novosphingobium*, which belongs to Proteobacteria, achieved distinctly higher association than other bacterial genera, demonstrating its indispensable role in bridging microbial communities and environmental factors. The same was true for *Fusarium*, affiliated with Ascomycota. It is noteworthy that four environmental factors (DOC, NH_4_^+^-N, NO_3_^−^-N, and TN) exhibited evidently strong correlations with microbial species. *Westerdykella*, *Novosphingobium*, and *Caulobacter* with the ability to degrade refractory organic compounds showed strongly negative associations with DOC, suggesting their crucial role in the degradation of refractory organics with the increased pretreatment degree. As the main identified denitrifying bacteria, *Kaistia* and *Chryseobacterium* revealed distinctly positive associations with DOC, which might be attributed to their feature of denitrification using carbon sources. *Selenomonas* and *Prevotella* were positively related to NH_4_^+^-N, which was largely due to their potential to participate in DNRA [60,61]. The strong negative association between *Xanthobacter* and NO_3_^−^-N or TN demonstrated the central role of this genus in NO_3_^−^-N reduction. These genera with relatively low abundance but high associations implied that it might be their roles in microbial networks or unique metabolic pathways rather than their abundance dominance that contributed to microbial denitrification and carbon source degradation [62].

## 4. Conclusions

The proper pretreatment of plant materials helps to improve denitrification performances and reduce the adverse effects of a PBS/corncob composite carbon source. The PBS/corncob composite with alkaline pretreatment promoted the enrichment of denitrifying microorganisms and reduced N_2_O emissions by regulating the ratios of ∑*nir*/∑*nos*. Microbial community analysis showed the bacterial community was responsible for denitrification and lignocellulose degradation, while the fungal community was mainly responsible for lignocellulose degradation. Some genera with low relative abundance might play important bonding roles in microbial networks. Overall, the polybutylene succinate/corncob composite with alkaline pretreatment could be a promising and eco-friendly carbon source for biological denitrification.

## Data Availability

Data are available upon request due to privacy and ethical restrictions.

## Supplementary Materials

The following supporting information can be downloaded at: https://www.mdpi.com/article/10.3390/polym15040801/s1, Figure S1: The variations in TN removal efficiency (%) in different SPD systems; Table S1: Primers of target genes used in qPCR analysis; Table S2: The results of ANOVA for the model (response: TN removal efficiency); Table S3: The optimized TN removal efficiency based on RSM; Table S4: The actual TN removal efficiency under optimized conditions; Table S5: Functional classification of major genera in bacterial and fungal communities. [63,64,65,66,67,68,69,70,71,72,73,74,75,76,77,78,79,80,81,82,83,84,85,86,87,88,89,90,91,92,93,94,95,96,97] of the references in the main text are from the supplementary material.
