# Supplementary material for: Improved Denitrification Performance of Polybutylene Succinate/Corncob Composite Carbon Source by Proper Pretreatment: Performance, Functional Genes and Microbial Community Structure
Source: Polymers (Basel). 2023 Feb 5;15(4):801. doi: 10.3390/polym15040801 (PMC9958998; doi:10.3390/polym15040801)
Supplement: Supplementary file 1 [file polymers-15-00801-s001.zip › polymers-2175032-supplementary.pdf]

Supplementary Materials

Improved denitrification performance of polybutylene succinate/corncob composite carbon source by proper pretreatment: performance, functional genes and microbial community structure

Zhongchen Yang, Yanhong Lou, Hong Pan, Hui Wang, Quangang Yang, Yuping Zhuge * and Jingying Hu

National Engineering Research Center for Efficient Utilization of Soil and Fertilizer Resources, College of Resources and Environment, Shandong Agricultural University, Tai'an 271018, China

* Correspondence: zhugeyp@sdau.edu.cn

Cover sheet

12 pages, 1 Figure, 5 Tables

Figure S1. The variations of TN removal efficiency (%) in different SPD systems

Table S1. Primers of target genes used in qPCR analysis

Table S2. The results of ANOVA for model (Response: TN removal efficiency)

Table S3. The optimized TN removal efficiency based on RSM

Table S4. The actual TN removal efficiency under optimized conditions

Table S5. Functional classification of major genera in bacterial and fungal communities

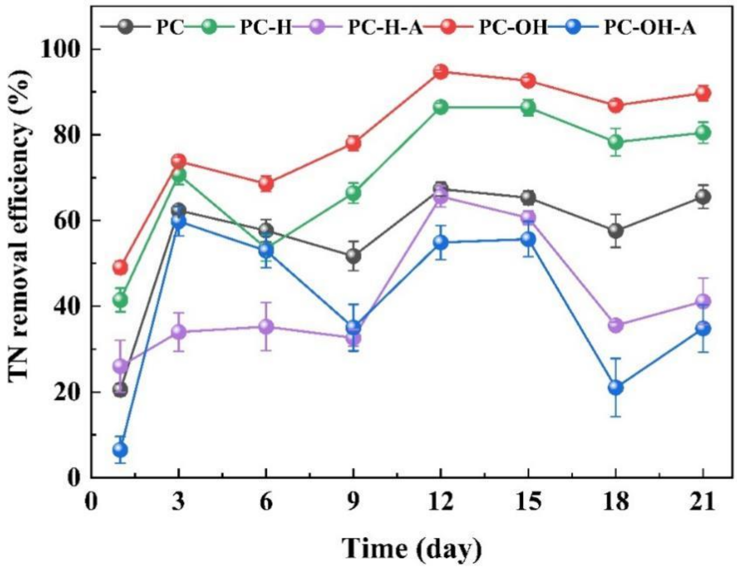

Figure S1. The variations of TN removal efficiency (%) in different SPD systems

Table S1. Primers of target genes used in qPCR analysis

Target gene	Primer	Primer sequence (5′-3′)	References
AOB-amoA	amoA-F	GGGGTTTCTACTGGTGGT	[1]
	amoA-R	CCCCTCKGSAAAGCCTTCTTC	
AOA-amoA	Arch-amoA-F	STAATGGTCTGGCTTAGACG	[2]
	Arch-amoA-R	GCGGCCATCCATCTGTATGT	
amx 16S rRNA	AMX809F	GCCGTAAACGATGGGCACT	[3]
	AMX1066R	AACGTCTCACGACACGAGCTG	
nrfA	6F	GAY TGC CAY ATG CCRAAA GT	[4]
	6R	GCB KCT TTY GCT TCRAAG TG	
narG	1960m2f	TA(CT)GT(GC)GGGCAGGA(AG)AAACTG	[5]
	2050m2r	CGTAGAAGAAGCTGGTGCTGTT	
napA	napAz3F	CGCGAACAAGCTGATGAAGG	[6]
	napAz3R	AAGATCATCGGGATGTCGGC	
nirK	nirK583F	TCATGGTGCTGCCGCGKGACGG	[7]
	nirK909R	GAACTTGCCGGTKGCCCAGAC	
nirS	nirScd3AF	GT(C/G)AACGT(C/G)AAGGA(A/G)AC(C/G)GG	[8]
	nirSR3cd	GA(C/G)TTCGG(A/G)TG(C/G)GTCTTGA	
nosZ (clade I)	nosZ-F	CGYTGTTCMTCGACAGCCAG	[9]
	nosZ1622R	CGSACCTTSTTGCCSTYGCG	
nosZ (clade II)	NosZ II-F	CTIGGICCIYTKCAYAC	[10]
	NosZ II-R	GCIGARCARAAITCBGTRC	
16S rRNA	338F	ACTCCTACGGGAGGCAGCAG	[11]
	518R	ATTACCGCGGCTGCTGG	

Table S2. The results of ANOVA for model (Response: TN removal efficiency)

Source	Sum of Squares	Mean Square	F-value	p-value	Note
Model	14,335.36	1791.92	375.42	< 0.0001	significant
A: Pretreatment degree	661.92	661.92	138.68	< 0.0001	
B: NO_3^--N concentration	1.82	1.82	0.3817	0.5539	
C: Temperature	4247.86	4247.86	889.95	< 0.0001	
AB	29.06	29.06	6.09	0.0389	
AC	350.25	350.25	73.38	< 0.0001	
A^2	557.87	557.87	116.88	< 0.0001	
C^2	45.42	45.42	9.51	0.0150	
A^2C	275.49	275.49	57.72	< 0.0001	
Residual	38.19	4.77			
Lack of Fit	19.70	4.92	1.07	0.4763	not significant
Pure Error	18.49	4.62			

Table S3. The optimized TN removal efficiency based on RSM

Number	Pretreatment degree	NO_3^--N concentration (mg L^{-1})	Temperature (°C)	TN removal efficiency (%)	Desirability	
1	0.531594841	14.99992049	24.99999492	91.7825482	0.907136944	Selected
2	0.541566294	14.99995107	24.99999287	91.77972237	0.90710501	
3	0.518194726	14.99996548	24.99998626	91.77798714	0.907085401	
4	0.556408009	14.99984071	24.9999679	91.76549526	0.906944234	
5	0.514431043	14.99975237	24.99695708	91.76101632	0.906893619	
6	0.516930336	14.9515668	24.999996	91.74704895	0.906735777	
7	0.574260425	14.999987	24.99927943	91.7299519	0.906542569	
8	0.527468769	14.91194198	24.99997122	91.72837717	0.906524773	
9	0.54289957	14.84224007	24.99996163	91.68507708	0.906035451	
10	0.602493796	14.99998752	24.99316717	91.61588318	0.905253511	
11	0.581349547	14.77395506	24.99999791	91.59027334	0.904964102	
12	0.54990473	14.6311415	24.99999658	91.55679224	0.904585741	
13	0.643922214	14.99996155	24.99998757	91.44373761	0.903308143	
14	0.556633342	14.99993213	24.92751287	91.43401442	0.903198264	
15	0.538691273	14.37808752	24.99999191	91.40883124	0.902913677	
16	0.571483361	14.14258607	24.99995836	91.25500702	0.901175353	
17	0.571134892	14.08987423	24.99996186	91.22567152	0.900843841	
18	0.521745988	14.99981381	24.86749317	91.17542616	0.900276033	
19	0.547471514	13.59111174	24.99998589	90.94494318	0.897671411	
20	0.545220706	13.48399064	24.99998414	90.88007444	0.896938348	
21	0.562533931	13.38600826	24.99997245	90.82996255	0.896372048	
22	0.40711032	14.99979014	24.8402934	90.64868183	0.894323447	
23	0.737856439	14.99999838	24.99998823	90.64331207	0.894262765	
24	0.536140053	12.68423273	24.99998361	90.38958976	0.891395522	
25	0.588984756	12.44909535	24.99998057	90.29902295	0.890372053	
26	0.625869035	12.29107571	24.99996633	90.16572413	0.888865681	

27	0.550287797	12.12128899	24.99998998	90.08416199	0.887943971
28	0.503814944	12.35112249	24.99998863	90.08149896	0.887913877
29	0.582464377	11.66744719	24.99998512	89.86825469	0.885504065
30	0.537712775	11.60357795	24.99998916	89.7449247	0.884110348
31	0.549742663	14.99978399	24.54704018	89.71082378	0.883724983
32	0.550840692	11.282162	24.99999565	89.59348068	0.882398923
33	0.610280841	11.09808593	24.99999352	89.56853388	0.882117006
34	0.595122963	10.9259106	24.99999981	89.47229051	0.881029388
35	0.569010042	10.77471684	24.99997091	89.34740508	0.879618093
36	0.609586527	9.929180195	24.99999965	88.95450807	0.875178077
37	0.423745332	11.33066875	24.99997582	88.84222025	0.873909145
38	0.676552939	9.602779035	24.99997867	88.75626537	0.872937794
39	0.539759657	9.786983078	24.99999179	88.66588098	0.871916386
40	0.170456189	14.9999345	24.99998358	88.30812975	0.867873542
41	0.689044695	8.41780587	24.99999387	88.19944105	0.866645283
42	0.670229352	7.933929541	24.99999543	87.99669529	0.864354111
43	0.594057722	8.063881781	24.99999538	87.921734	0.863506995
44	0.628188755	7.504604196	24.99999468	87.73744623	0.861424412
45	0.638490472	7.396737572	24.99999929	87.70757093	0.8610868
46	0.478358006	8.849949819	24.99999785	87.64351675	0.860362942
47	0.664457973	6.688452474	24.9999937	87.41265394	0.857754028
48	0.690335763	6.551281806	24.99999757	87.37215836	0.857296399
49	0.727193388	6.569808941	24.99999038	87.35178189	0.857066131
50	0.681372027	6.21929638	24.9999975	87.21812825	0.855555749
51	0.72439268	5.86375389	24.99998327	87.06915055	0.853872195
52	0.699363174	5.764720489	24.99997352	87.03154422	0.853447217
53	0.70553712	5.642505755	24.99998178	86.9819027	0.852886232
54	0.725226957	5.645578283	24.99999699	86.97986697	0.852863227
55	0.688104044	5.557073965	24.9999848	86.92964767	0.852295713
56	0.762518755	5.596190498	24.99998612	86.89848073	0.851943505

57	0.724185032	5.262026788	24.99999635	86.82440369	0.851106381
58	0.726995289	5.146310342	24.99998914	86.77668726	0.850567152
59	0.694518245	5.135030111	24.99998494	86.75260842	0.850295044
60	0.52532684	6.694087824	24.99998673	86.69511565	0.849645335
61	0.760263002	5.000025213	24.98830257	86.63062766	0.848916574
62	0.070746843	14.9999879	24.99999923	86.12256274	0.843175079
63	0.239162787	5.000053866	24.99999378	80.4278857	0.778821174

Table S4. The actual TN removal efficiency under optimized conditions

**Number**	**Pretreatment degree**	**NO_3^--N concentration ($mg\ L^{-1}$)**	**Temperature (^oC)**	**TN removal efficiency (%)**	Average **TN removal efficiency (%)**
1	1	15	25	90.09	
2	1	15	25	88.02	
3	1	15	25	89.72	89.73
4	1	15	25	92.84	
5	1	15	25	88.98	

Table S5. Functional classification of major genera in bacterial and fungal communities

Microbial Functions	Bacterial community	Fungal community
Denitrification	Curvibacter [12], Allorhizobium-Neorhizobium-Pararhizobium-Rhizobium [13], Kaistia [14], Enterobacter [15], Sphingomonas [16], Chryseobacterium [17], Selenomonas [18], Ralstonia [19], Bosea [20], Haliangium [21], Cupriavidus [22], Burkholderia-Caballeronia-Paraburkholderia [23], Variovorax [24], Herbaspirillum [25], Diaphorobacter [26], Xanthobacter [27], Paludibacter [28]	
Lignocellulose degradation	Dysgonomonas [29], Reyranella [30], Cellulomonas [31], Propionispira [32], Pleomorphomonas [33], Novosphingobium [34]	Chaetomium [35], Fusarium [36], Trichoderma [37], Paracremonium [38], Humicola [39], Apiotrichum [40], Staphylotrichum [41], Ascobolus [42]

References

1. Rotthauwe, J. H.; Witzel, K. P.; Liesack, W. The ammonia monooxygenase structural gene amoA as a functional marker: molecular fine-scale analysis of natural ammonia-oxidizing populations. *Appl. Environ. Microbiol.* **1997**, *63*, 4704-4712.
2. Francis, C. A.; Roberts, K. J.; Beman, J. M.; Santoro, A. E.; Oakley, B. B. Ubiquity and diversity of ammonia-oxidizing archaea in water columns and sediments of the ocean. *PNAS* **2005**, *102*, 14683-14688.
3. Tsushima, I.; Kindaichi, T.; Okabe, S. Quantification of anaerobic ammonium-oxidizing bacteria in enrichment cultures by real-time PCR. *Water Res.* **2007**, *41*, 785-794.
4. Takeuchi, J. Habitat segregation of a functional gene encoding nitrate ammonification in estuarine sediments. *Geomicrobiol. J.* **2006**, *23*, 75-87.
5. López-Gutiérrez, J. C.; Henry, S.; Hallet, S.; Martin-Laurent, F.; Catroux, G.; Philippot, L. Quantification of a novel group of nitrate-reducing bacteria in the environment by real-time PCR. *J. Microbiol. Methods* **2004**, *57*, 399-407.
6. Bru, D.; Sarr, A.; Philippot, L. Relative abundances of proteobacterial membrane-bound and periplasmic nitrate reductases in selected environments. *Appl. Environ. Microbiol.* **2007**, *73*, 5971-5974.
7. Liu, X.; Tiquia, S. M.; Holguin, G.; Wu, L.; Nold, S. C.; Devol, A. H.; Luo, K.; Palumbo, A. V.; Tiedje, J. M.; Zhou, J. Molecular diversity of denitrifying genes in continental margin sediments within the oxygen-deficient zone off the Pacific coast of Mexico. *Appl. Environ. Microbiol.* **2003**, *69*, 3549-3560.
8. Kandeler, E.; Deiglmayr, K.; Tscherko, D.; Bru, D.; Philippot, L. Abundance of narG, nirS, nirK, and nosZ genes of denitrifying bacteria during primary successions of a glacier foreland. *Appl. Environ. Microbiol.* **2006**, *72*, 5957-5962.
9. Li, X.; Wang, H.; Hu, C.; Yang, M.; Hu, H.; Niu, J. Characteristics of biofilms and iron corrosion scales with ground and surface waters in drinking water distribution systems. *Corros Sci* **2015**, *90*, 331-339.
10. Jones, C. M.; Graf, D. R.; Bru, D.; Philippot, L.; Hallin, S. The unaccounted yet abundant nitrous oxide-reducing microbial community: a potential nitrous oxide sink. *The ISME journal* **2013**, *7*, 417-426.
11. Muyzer, G.; De Waal, E. C.; Uitterlinden, A. Profiling of complex microbial populations by denaturing gradient gel electrophoresis analysis of polymerase chain reaction-amplified genes coding for 16S rRNA. *Appl. Environ. Microbiol.* **1993**, *59*, 695-700.
12. Zielińska, M.; Rusanowska, P.; Jarząbek, J.; Nielsen, J. L. Community dynamics of denitrifying bacteria in full-scale wastewater treatment plants. *Environ. Technol.* **2016**, *37*, 2358-2367.
13. He, X.; Zhang, S.; Jiang, Y.; Li, M.; Yuan, J.; Wang, G. Influence mechanism of filling ratio on solid-phase denitrification with polycaprolactone as biofilm carrier. *Bioresour. Technol.* **2021**, *337*, 125401.
14. Wang, C.; Zhu, G.; Wang, Y.; Wang, S.; Yin, C. Nitrous oxide reductase gene (nosZ) and N_2O reduction along the littoral gradient of a eutrophic freshwater lake. *J Environ Sci* **2013**, *25*, 44-52.
15. Zhou, B.; Duan, J.; Xue, L.; Zhang, J.; Yang, L. Effect of plant-based carbon source supplements on denitrification of synthetic wastewater: focus on the microbiology. *Environ. Sci. Pollut. Res.* **2019**, *26*, 24683-24694.
16. Li, H.; Zhou, Z.; Liu, Q.; Dong, H.; Duan, Y.; Li, C.; Zhang, J.; Tan, H. Biological denitrification in high salinity wastewater using semen litchi as a carbon source. *Rsc Advances* **2015**, *5*, 92836-92842.
17. Kundu, P.; Pramanik, A.; Dasgupta, A.; Mukherjee, S.; Mukherjee, J. Simultaneous heterotrophic nitrification and aerobic denitrification by Chryseobacterium sp. R31 isolated from abattoir wastewater. *BioMed Res. Int.* **2014**, 2014.
18. Greening, C.; Geier, R.; Wang, C.; Woods, L. C.; Morales, S. E.; McDonald, M. J.; Rushton-Green, R.; Morgan, X. C.; Koike, S.; Leahy, S. C.; Kelly, W. J.; Cann, I.; Attwood, G. T.; Cook, G. M.; Mackie, R. I. Alternative hydrogen uptake pathways suppress methane production in ruminants. *bioRxiv* **2018**, 486894.
19. Xiao, Y.; Zheng, Y.; Wu, S.; Yang, Z. H.; Zhao, F. Bacterial community structure of autotrophic denitrification biocathode by 454 pyrosequencing of the 16S rRNA gene. *Microb. Ecol.* **2015**, *69*, 492-499.
20. Miller, M. N.; Dandie, C. E.; Zebarth, B. J.; Burton, D. L.; Goyer, C.; Trevors, J. T. Influence of carbon amendments on soil denitrifier abundance in soil microcosms. *Geoderma* **2012**, *170*, 48-55.
21. Wang, H.; Sun, Y.; Zhang, L.; Wang, W.; Guan, Y. Enhanced nitrogen removal and mitigation of nitrous oxide emission potential in a lab-scale rain garden with internal water storage. *J. Water Process. Eng.* **2021**, *42*, 102147.

22. Xie, C. H.; Yokota, A. Reclassification of [Flavobacterium] ferrugineum as Terrimonas ferruginea gen. nov., comb. nov., and description of Terrimonas lutea sp. nov., isolated from soil. *Int. J. Syst. Evol. Microbiol.* **2006**, *56*, 1117-1121.
23. Hetz, S. A.; Horn, M. A. *Burkholderiaceae* are key acetate assimilators during complete denitrification in acidic cryoturbated peat circles of the arctic tundra. *Front. Microbiol.* **2021**, *12*, 628269.
24. Liu, D.; Li, J.; Li, C.; Deng, Y.; Zhang, Z.; Ye, Z.; Zhu, S. Poly (butylene succinate)/bamboo powder blends as solid-phase carbon source and biofilm carrier for denitrifying biofilters treating wastewater from recirculating aquaculture system. *Sci. Rep.* **2018**, *8*, 1-12.
25. Bonato, P.; Batista, M. B.; Camilios-Neto, D.; Pankievicz, V. C.; Tadra-Sfeir, M. Z.; Monteiro, R. A.; Pedrosa, F. O.; Souza, E. M.; Chubatsu, L. S.; Wassem, R.; Rigo, L. U. RNA-seq analyses reveal insights into the function of respiratory nitrate reductase of the diazotroph Herbaspirillum seropedicae. *Environ. Microbiol.* **2016**, *18*, 2677-2688.
26. Wang, J.; Chu, L. Biological nitrate removal from water and wastewater by solid-phase denitrification process. *Biotechnol. Adv.* **2016**, *34*, 1103-1112.
27. King, G. M. Nitrate-dependent anaerobic carbon monoxide oxidation by aerobic CO-oxidizing bacteria. *FEMS Microbiol. Ecol.* **2006**, *56*, 1-7.
28. Qiu, Y. L.; Kuang, X. Z.; Shi, X. S.; Yuan, X. Z.; Guo, R. B. Paludibacter jiangxiensis sp. nov., a strictly anaerobic, propionate-producing bacterium isolated from rice paddy field. *Arch. Microbiol.* **2014**, *196*, 149-155.
29. Soares, L. A.; Silva Rabelo, C. A. B.; Sakamoto, I. K.; Silva, E. L.; Varesche, M. B. A. Screening and bioprospecting of anaerobic consortia for biofuel production enhancement from sugarcane bagasse. *Appl. Biochem. Biotechnol.* **2020**, *190*, 232-251.
30. Song, W. F.; Wang, J. W.; Yan, Y. C.; An, L. Y.; Zhang, F.; Wang, L.; Xu, Y.; Tian, M. Z.; Nie, Y.; Wu, X. L. Shifts of the indigenous microbial communities from reservoir production water in crude oil- and asphaltene-degrading microcosms. *Int. Biodeterior. Biodegrad.* **2018**, *132*, 18-29.
31. Rusznyak, A.; Toth, E. M.; Schumann, P.; Spröer, C.; Makk, J.; Szabo, G.; Vladar, P.; Marialigeti, K.; Borsodi, A. K. Cellulomonas phragmiteti sp. nov., a cellulolytic bacterium isolated from reed (Phragmites australis) periphyton in a shallow soda pond. *Int. J. Syst. Evol. Microbiol.* **2011**, *61*, 1662-1666.
32. Xafenias, N.; Anunobi, M. O.; Mapelli, V. Electrochemical startup increases 1, 3-propanediol titers in mixed-culture glycerol fermentations. *Process Biochem* **2015**, *50*, 1499-1508.
33. Chang, J. J.; Lin, J. J.; Ho, C. Y.; Chin, W. C.;Huang, C. C. Establishment of rumen-mimic bacterial consortia: a functional union for bio-hydrogen production from cellulosic bioresource. *Int. J. Hydrog. Energy* **2010**, *35*, 13399-13406.
34. Wang, J.; Wang, C.; Li, J.; Bai, P.; Li, Q.; Shen, M.; Li, R.; Li, T.; Zhao, J. Comparative genomics of degradative Novosphingobium strains with special reference to microcystin-degrading Novosphingobium sp. THN1. *Front. Microbiol.* **2018**, *9*, 2238.
35. Darwish, A. M.; Abdel-Azeem, A. M. Chaetomium enzymes and their applications, Recent Developments on Genus Chaetomium. *Springer*, **2020**, 241-249.
36. Huang, Y.; Busk, P. K.; Lange, L. Cellulose and hemicellulose-degrading enzymes in *Fusarium commune* transcriptome and functional characterization of three identified xylanases. *Enzyme Microb. Technol.* **2015**, *73*, 9-19.
37. Novy, V.; Nielsen, F.; Seiboth, B.; Nidetzky, B. The influence of feedstock characteristics on enzyme production in Trichoderma reesei: a review on productivity, gene regulation and secretion profiles. *Biotechnol. Biofuels* **2019**, *12*, 1-16.
38. Qi, W.; Taherzadeh, M. J.; Ruan, Y.; Deng, Y.; Chen, J. S.; Lu, H. F.; Xu, X. Y. Denitrification performance and microbial communities of solid-phase denitrifying reactors using poly (butylene succinate)/bamboo powder composite. *Bioresour. Technol.* **2020**, *305*, 123033.
39. S Steindorff, A. S.; Serra, L. A.; Formighieri, E. F.; de Faria, F. P.; Poças-Fonseca, M. J.; de Almeida, J. R. M. Insights into the Lignocellulose-Degrading Enzyme System of *Humicola grisea* var. *thermoidea* Based on Genome and Transcriptome Analysis. *Microbiol. Spectr.* **2021**, *9*, e01088-21.
40. Štursová, M.; Žifčáková, L.; Leigh, M. B.; Burgess, R.; Baldrian, P. Cellulose utilization in forest litter and soil: identification of bacterial and fungal decomposers. *FEMS Microbiol. Ecol.* **2012**, *80*, 735-746.
41. Ali, A.; Ellinger, B.; Brandt, S. C.; Betzel, C.; Rühl, M.; Wrenger, C.; Schlüter, H.; Schäfer, W.; Brognaro, H.; Gand, M. Genome and Secretome Analysis of Staphylotrichum longicolleum DSM105789 Cultured on Agro-Residual and Chitinous Biomass. *Microorganisms* **2021**, *9*, 1581.
42. Tian, X.; Yang, T.; He, J.; Chu, Q.; Jia, X.; Huang, J. Fungal community and cellulose-degrading genes in the composting process of Chinese medicinal herbal residues. *Bioresour. Technol.* **2017**, *241*, 374-383.
